# Intertemporal meditation regulates time perception and emotions: an exploratory fNIRS study

**DOI:** 10.1093/scan/nsaf080

**Published:** 2025-08-01

**Authors:** Feng Xiao, Qimei Peng, XiaoTian Wang

**Affiliations:** Department of Applied Psychology, School of Humanities and Social Science, The Chinese University of Hong Kong (Shenzhen), Shenzhen 518172, China; Department of Applied Psychology, School of Humanities and Social Science, The Chinese University of Hong Kong (Shenzhen), Shenzhen 518172, China; Department of Applied Psychology, School of Humanities and Social Science, The Chinese University of Hong Kong (Shenzhen), Shenzhen 518172, China

**Keywords:** intertemporal meditation, mindfulness meditation, episodic future thinking, emotion regulation, time perception, fNIRS

## Abstract

Mental time travel enables humans to project life episodes into the future, shaping present behaviour through episodic future thinking. This study explored the effects of repeated mental time travel from the present to the end of life on the time perception and emotional experiences of young adults. Unlike previous studies that focused solely on a single moment (present or death), we introduced intertemporal meditation, which combines mindfulness meditation and episodic future thinking. Using a within-subject design combined with fNIRS recording, we compared pre-meditation with post-meditation measures in both mindfulness and intertemporal meditation conditions. Behavioural results showed that intertemporal meditation shortened time perception and made the participants feel more peaceful and less anxious. The fNIRS results revealed that intertemporal meditation resulted in higher overall brain activations than mindfulness meditation. Hemodynamic measures revealed significant left-lateralized activations in brain regions associated with emotion regulation and cognitive control, particularly the dorsolateral prefrontal cortex, anterior prefrontal cortex, and orbitofrontal cortex. In addition, right-lateralized activations in Broca’s area and middle temporal gyrus indicated enhanced processing of social cues and autobiographical memory processing. These findings underscore the adaptive utility of intertemporal meditation in emotion regulation and its potential to improve cognitive control functions.

## Introduction

*Mental time travel* is a unique mental faculty that evolved through human cognitive evolution (Tulving 2002). This capacity enables us to project consciousness forward to the future and construct anticipated scenarios (Suddendorf and Corballis 2007, Suddendorf et al. 2009). One of its primary forms is *episodic future thinking*, a goal-directed mental simulation that connects the present events with imagined future events and the present self with the imagined future self (Buckner and Carroll 2007, Hassabis et al. 2007). This flexible foresight helps develop adaptive strategies for fine-tuning human behaviours and allows us to address time-related dilemmas in life (Suddendorf 2006). In addition, by constructing vivid mental imagery, episodic future thinking elicits *anticipatory emotions*, which serve as informative cues at the time of decision-making (Wang et al. 2021, 2022).

An extreme case of episodic future thinking is to contemplate one’s own death or imagine the episodes at the end of one’s life. The impact of death awareness is evident in time perception and future-related choices. Thinking about one’s death changed the time perception of the young participants, who felt that time passed faster (Wang et al. 2019). The participants who experienced the death of close ones from cancer (Liu and Aaker 2007) or thought about their own death (Kelley and Schmeichel 2015, Wang et al. 2019) exhibited higher preferences for future-oriented rewards. Conversely, other studies indicated that contemplating death enhanced desires for short-term goals (Gailliot et al. 2006, Hart et al. 2010). Terror management theory (TMT) explains this inconsistency through distal and proximal defences (Greenberg et al. 1986, Pyszczynski et al. 2000, Dechesne et al. 2003). According to TMT, the awareness that one’s death is inevitable (also termed *mortality salience*) induces anxiety and fear. As a distal defence, individuals adopt future-oriented behaviours (e.g. elevating self-esteem) to manage these unsettling emotions. In contrast, proximal defences prompt present-focused behaviours to alleviate the immediate negative emotions elicited by thoughts of death.

We argue that the behavioural effects of death awareness are not merely due to contemplating the moment of death but in reflecting on the time passage from the present to the end of life. Both distal and proximal defences treat death as an intimidating outcome, focusing solely and passively on the inevitable termination of one’s life (Mroczek and Almeida 2004, Navarrete and Fessler 2005, Boyer 2007). However, life is a fluid process, and time continuously moves forward. The perception of temporal distance to the end of life provides information for motivational changes and adaptive intertemporal decisions (Carstensen et al. 2003, Wang et al. 2019).

We propose a new perspective of ‘adaptive time management’ to explain how individuals regulate temporal priorities in accordance with survival and reproductive demands across the lifespan (Wang et al. 2019, Lu et al. 2023, Wang and Wang 2024). In this view, time perception interacts with emotional experience to guide adaptive decision-making. In contrast to the passive, defensive mechanisms described by TMT, adaptive time management emphasizes active, flexible adjustment of time-related goals—balancing short- and long-term rewards as well as social utility and emotional needs depending on life stage (Hare et al. 2005, Haselton et al. 2009, Lu et al. 2023). For example, socioemotional selectivity theory (SST) posits that older adults, perceiving a limited future time horizon, tend to prioritize present-oriented emotional satisfaction over distant instrumental goals (Urry and Gross 2010, Carstensen et al. 2011, Brose et al. 2015).

Equipped with the ability of mental time travel, humans are sensitive to the distance from the present time to the end of life and regulate their social and emotional well-being accordingly. From this perspective, we developed *intertemporal meditation*, a novel approach utilizing mental time travel from the present time to the end of life to regulate the subject’s time perception and emotional experiences over a few repeated mental time travels. It combines elements of mindfulness with episodic end-of-life reflection and may hold practical value for therapeutic interventions, particularly in palliative care or grief-focused psychotherapy where existential concerns are central (Yalom 2020, Xunlin et al. 2020). For young adults, typically distant from the end of life, intertemporal meditations should generate a feeling that ‘time flies’ or ‘life is short’ and thus accelerate time perception. In addition, we expect that intertemporal meditation will induce anticipatory emotions during the mental time travel. These emotional experiences are expected to be not only negative but also adaptive.

Previous studies on mindfulness meditation practice and time perception yielded mixed results. Depending on the anxiety level and attention regulation, mindfulness meditation may either shorten (Droit-Volet et al. 2018) or lengthen subjective time perception (Kramer et al. 2013). Although the exact mechanism of mindfulness meditation on time perception remains to be determined, we expect that intertemporal meditation would shorten time perception due to its dynamic nature of mental travels.

It is well-known that mindfulness meditation can regulate emotional experiences by fostering a present temporal focus (Kabat-Zinn and Hanh 2009, Hölzel et al. 2011, Lutz et al. 2014, Kober et al. 2019). In a pilot study, we recruited young adults to compare intertemporal with mindfulness meditation. Our results suggested that young adults exhibited accelerated time perception after intertemporal meditation. The dominant arousal emotions during intertemporal meditation were *peace*, *relaxation*, and *sadness*, rather than *anxiety* and *fear* indicated by TMT (Greenberg et al. 1986). These findings suggested that intertemporal meditation accelerates time perception and induces adaptive emotional responses.

In addition, the present study also examined the neural mechanism underlying these behavioural changes. Previous studies have shown that cognitive control is one of the core executive functions for regulating emotional experiences (Diamond 2013, Timpano and Schmidt 2013). During emotion regulation, brain regions related to executive functions, including the dorsolateral prefrontal cortex (DLPFC, Brodmann area 9 and 46) and anterior prefrontal cortex (aPFC, Brodmann area 10), are activated to down-regulate the excitability of the amygdala so as to stabilize emotional states (Kanske et al. 2011, Buhle et al. 2014). Moreover, the orbitofrontal cortex (OFC, Brodmann area 11) plays a pivotal role in impulse control (Ochsner et al. 2002). Specifically, the OFC contributes to the appraisal of emotional stimuli, inhibition of inappropriate responses, and generation of behavioural strategies in response to negative emotional cues (Kringelbach and Rolls 2004, Rolls 2019). These cortical regions thus contribute to enhancing cognitive control and regulating emotional responses.

Based on the above analysis, the present study proposed three *hypotheses*:

- Participants after intertemporal meditation practice would feel a faster passage of time due to repeated mental travels, whereas after mindfulness meditation would not. That is, the subjective passage of time will be faster due to mental time travel to the end of one’s life.
- Intertemporal meditation would induce adaptive rather than defensive emotional responses. The anticipatory emotions induced by death awareness or meditation about the end-of-life episodes will be not only negative but also adaptive and uplifting due to simulated confrontations with the end of one’s life.
- Time perception in intertemporal meditation could better regulate young adults’ emotional experiences than mindfulness meditation by potentially enhancing cognitive control capacities.

## Materials and Methods

### Participants

We recruited 70 young adults (29 males and 41 females) from the Chinese University of Hong Kong (Shenzhen). The sample size was estimated based on our preliminary pilot study. All the materials presented in the experiment were in Chinese. The average age of the participants was 21.72 ± 3.22 years. There was no significant age difference between males (*M *= 22.45, *SD *= 3.58) and females (*M *= 21.10, *SD *= 2.76).

One day before the experiment, participants were instructed to abstain from consuming any substances known to affect attention for at least two hours before the start of the experiment, including alcohol, tea, coffee, and cigarettes. Eligible participants read and signed an informed consent form before the experiment began. At the end of the experiment, researchers debriefed participants, and they received either academic credits or monetary compensation for their participation.

We strictly followed the guidelines of the Institutional Ethics Committee from the Chinese University of Hong Kong (Shenzhen) and received prior approval for the experiment (EF20211116001).

### Intertemporal and mindfulness meditations

A female experimenter trained in meditation practice narrated the audio instructions for both meditations before the experiment. Instructions for intertemporal and mindfulness meditations were identical, except that the former encompassed the mental time travel from the present to the end-of-life moment, and the latter focused on the present moment (Table S.1, see online supplementary material for a colour version of this table). Through the recorded audio instructions, our version of mindfulness meditation emphasized its aspects of internal awareness of feelings and thoughts and focused attention (Kristeller 2007).

**Table 1.** Regression of emotional experience on gender and meditation type.

	Gender		Meditation type		Interaction effects			
Emotion	*β*	*P*	*β*	*P*	*β*	*P*	*R*²	*P* (model)
Thrill	15.78	.012*	−0.24	.965	−12.53	.151	.069	.045*
Peace	10.39	.117	2.71	.647	2.93	.753	.062	.064
Anxiety	−2.94	.670	2.18	.727	−9.22	.344	.031	.329
Joy	12.73	.072	−5.06	.423	−4.20	.671	.057	.087
Fear	−3.04	.641	9.05	.126	1.87	.841	.043	.188
Sadness	12.38	.070	15.59	.012*	−1.57	.870	.129	.002**
Relaxation	8.18	.236	−12.85	.037*	6.84	.478	.090	.016*

This table presents the results of linear regressions predicting self-reported emotional experience based on gender (male vs. female), meditation type (intertemporal vs. mindfulness), and their interaction. Asterisks indicate the statistical significance of predictors or models.

* *P* < .05.

** *P* < .01.

**Figure 1.**
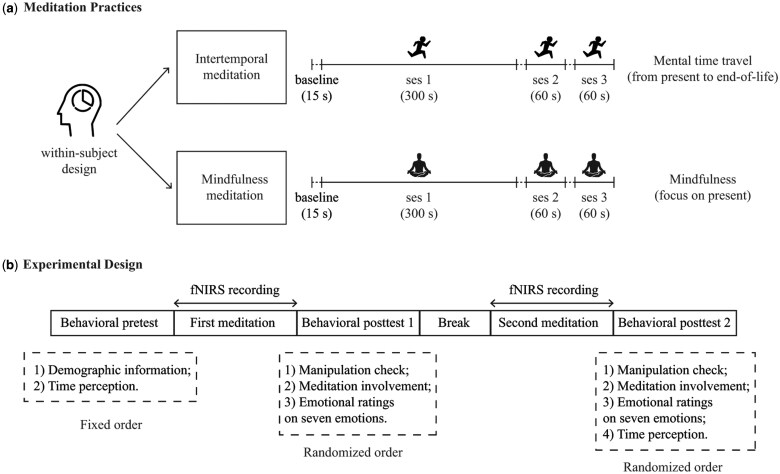
Procedural diagrams of meditation practices and experimental design. (a) Each type of meditation practice included three sessions. During intertemporal meditation, participants focused on the mental time travel process (i.e. from the present to the end of life). In contrast, during mindfulness meditation, participants focused on the present moment. (b) Behavioural measures included one pre-meditation test and two post-meditation tests. Self-reported emotional experiences (emotional ratings) were recorded in the two post-meditation tests. Time perception was measured in the pre-meditation test and the second post-meditation test.

The studies that have investigated the temporal effects of mindfulness meditation typically require the participants to follow recorded audio instructions to focus their attention moment-to-moment on the way they breathed (breathing exercise) or on the different parts of their body (body-scan exercise) (e.g. Kramer et al. 2013, Droit-Volet and Heros 2017). In the present study, participants followed the instructions to practice meditation in a quiet, dimly lit room with eyes closed. Each meditation practice lasted about seven minutes over three sessions (see Fig. 1a). A bell ring indicated the end of each session. The participants then opened their eyes and took a break between the sessions. The first 15 seconds of each session served as a baseline period for recording hemodynamic responses while the participants sat quietly with their eyes closed. In the first session (300 seconds), participants followed each audio instruction to meditate through body scanning and breathing exercises. Subsequently, participants mentally travelled gradually forward from the present moment to the end moment of life during intertemporal meditation, whereas they focused on the present moment during mindfulness meditation. Instructions guided participants to experience and feel this mental travel. The following two sessions repeated the meditation exercises with a shorter duration (60 seconds each). Through the sessions of intertemporal meditation, the participants mentally travelled from the present to the end of life and then back to the present to start another mental trip. It was like a mental exercise of ‘out-and-back trails’.

### Study design and behavioural measures

We employed a within-subject experimental design for both meditations with the order of practice randomized. Each participant underwent one pre-meditation test and two sets of post-meditation tests, one for mindfulness and one for intertemporal exercises (see Fig. 1b).

Participants conducted the pre-meditation test onsite via the Credamo platform (an equivalent of MTurk). We collected basic information in the pre-meditation test, including their gender, age, head circumference, dominant hand, and meditation practice experiences, mental illness and medication history, and time perception. To avoid possible initiation or expectation effects, we chose not to measure emotional experiences in the pre-meditation test, focusing on comparing the emotional profiles post-meditation between the types of meditation and changes over the three sessions.

For the time perception measure, we chose to use a more extended time unit in years to be consistent with the range of mental travel within the lifespan (Wang et al. 2019). Adopted from Zauberman et al. (2009), we asked the participants to rate how long or how short they perceive a 10-year period on a Likert scale from 1 (*Very short*) to 100 (*Very long*). To minimize task burden and potential carryover effects during the meditation intervention, we assessed time perception only at two points: once at the pre-meditation test and once after the second meditation intervention (either intertemporal or mindfulness, depending on the counterbalanced order).

After each meditation, participants completed post-meditation tests displayed by E-prime software (version 3.0) with the task order randomized. In both posttests, participants provided ratings for seven emotions (e.g. *Please rate how strongly you feel thrilled during your meditation*), including *thrill*, *peace*, *joy*, *relaxation*, *anxiety*, *fear*, and *sadness*, on a Likert scale from 1 (*Not at all*) to 100 (*Very strong*). We chose these emotions based on our pilot study. Additionally, participants rated their level of involvement in the meditation on a Likert scale from 1 (*Totally out of meditation*) to 100 (*Totally involved in meditation*). Participants had to specify the correct meditation type (intertemporal or mindfulness meditation) for a manipulation check.

### Hemodynamic response monitoring

We used NIRSport2 (NIRx, Germany) to record hemodynamic responses during both meditation practices (see Fig. 1b). The device consists of 16 sources and 16 detectors and records optical density data at a sampling rate of 5.1 Hz. It measures deoxygenated and oxygenated haemoglobin levels at wavelengths of 760 nm and 850 nm, respectively.

**Figure 2.**
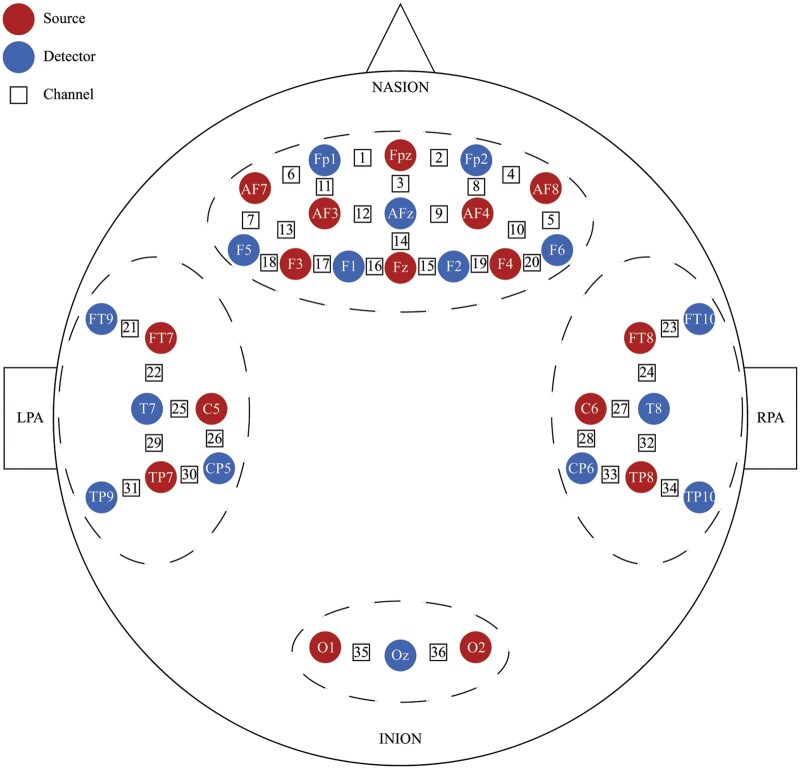
fNIRS montage. Circles marked "Source" denote the sources, circles marked "Detector" denote the detectors, and squares denote the channels. The dashed areas are referred to as the ROIs. The brain regions based on the 10–10 system were displayed for sources and detectors. The distance between each pair of sources and detectors that constituted a channel was ∼30 mm.

Figure 2 displays the setup of fNIRS optodes and channels. To ensure a good depth sensitivity and signal-to-noise ratio (SNR), the source-detector distance for each channel was within about 30 mm (Calderon-Arnulphi et al. 2009). A montage of 36 channels was created using NIRSite (version 2.0.1.0), comprising 20 channels over the prefrontal lobe, 14 over the temporal lobe (7 channels for each hemisphere), and two channels over the occipital lobe. These optodes were positioned according to the EEG 10-10 system.

The toolboxes to perform spatial registration and transform the coordinates of the optodes and channels from the montage to the MNI space via MATLAB (version R2013b) included the SPM8 and NIRS-SPM (version NIRS-SPM_V4_r1) (Ye et al. 2009). Based on the MNI coordinates, we assigned each channel to its corresponding Brodmann area (BA) with the largest overlap. On average, there was a 74% anatomical overlap probability across all channels (ranging from 34% to 100%). A total of 12 different Brodmann areas were covered, including BA9 (DLPFC), BA10 (aPFC), BA11 (OFC), BA45, and BA46 (DLPFC) in the prefrontal lobe; BA2L, BA20L, BA21 (middle temporal gyrus, MTG), BA22, BA37R, and BA40R in the temporal lobe; and BA17 in the occipital lobe. Table S.2 (see online supplementary material for a colour version of this table) displays the coordinates from the 10-10 system, MNI space, and corresponding Brodmann areas for each channel.

**Table 2.** Regression of emotional experience on time perception and meditation type.

	Δ Time perception		Meditation type		Interaction effects			
Emotion	*β*	*P*	*β*	*P*	*β*	*P*	*R*²	*P* (model)
Thrill	0.15	.605	−15.37	.025*	−0.17	.630	0.107	.113
Peace	−0.36	.239	10.26	.153	0.51	.179	0.055	.400
Anxiety	0.07	.844	−1.79	.824	0.25	.549	0.045	.492
Joy	0.25	.439	−18.35	.018*	−0.13	.748	0.149	.038*
Fear	0.05	.880	6.42	.419	0.45	.282	0.076	.246
Sadness	−0.74	.035*	25.07	.003**	1.11	.012*	0.193	.011*
Relaxation	0.10	.773	−7.46	.354	−0.11	.799	0.020	.791

Table 2 presents the results of multiple linear regressions predicting self-reported emotional experience based on the changes in time perception (post-meditation minus pre-meditation), meditation type (intertemporal vs. mindfulness), and their interaction. Asterisks indicate the statistical significance of predictors or models.

* *P* < .05.

** *P* < .01.

### Hemodynamic data preprocessing

We preprocessed the hemodynamic data using MATLAB (version R2017b) with the Homer3 toolbox (version 1.80.2) (Huppert et al. 2009). The data processing consisted of eight steps: (i) prune raw data to remove channels with extreme signals using *hmrR_PruneChannels* function *(dRange = 0.01 to 1, SNRthresh = 2, SDrange = 25 to 40)*, (ii) convert the input intensity data into optical density format using *hmrR_Intensity2OD* function, (iii) use *hmrR_MotionArtifactByChannel* function (*tMotion = 0.5, tMask = 1, STDEVthresh = 5, AMPthresh = 0.05*) to identify motion artifacts and remove them through wavelet transformation using *hmrR_MotionCorrectWavelet* function (*iqr = 1.5, turnon = 1*), (iv) apply a low-pass frequency filter to reduce high-frequency noise using *hmrR_BandpassFilt: Bandpass_Filter_OpticalDensity* function (*hpf = 0, lpf = 0.08*), (v) convert the optical density data into blood concentration format using *hmrR_OD2Conc* function (*ppf = [1.0 1.0]*), (vi) calculate the changes in oxygenated haemoglobin (HbO) and deoxygenated haemoglobin (HbR) using the modified Beer-Lambert law (mBLL) with a differential path factor (DPF) of 7.25 (760 nm) and 6.38 (850 nm), (vii) apply a correlation-based signal enhancement method to reduce motion artifacts again to improve concentration changes by using the *hmrR_MotionCorrectCbsi* function (*turnon = 1*), and (viii) extract the hemodynamic data from each meditation session using *hmrR_BlockAvg: Block_Average_on_Concentration_Data* function (session 1: *trange = −15 to 300*, session 2 & 3: *trange = −15 to 60*).

### Hemodynamic amplitude-based analysis

After the above preprocessing, we utilized in-house scripts in MATLAB (version R2017b) to analyse the hemodynamic data. We employed an amplitude-based analysis to detect brain regional activation and assessed the activation differences between the two meditation conditions (Gervain et al. 2011, Mandrick et al. 2013).

The participants’ data at each temporal point were averaged to integrate hemodynamic changes for every valid channel. We compared the changes in HbO and HbR, and selected HbO for further analysis due to its higher SNR (Tachtsidis and Scholkmann 2016, Pinti et al. 2020). Using the first 15 seconds before the onset of meditation as the baseline period for each session, we calculated the relative changes of HbO (unit: μM). To analyse the data, we integrated the relative changes in HbO from each session during meditation. We extracted HbO changes of the first session (300 seconds), the first session plus the first repetition (360 seconds), and the first session plus two repetitions (420 seconds). This approach allowed us to reveal the accumulated effects as each type of meditation practice progressed over the three sessions.

## Results

### Behavioural results

*Data inclusion for analysis.* We included 67 copies of behavioural data (27 males and 40 females) with an average age of 21.72 years for analysis. During data preprocessing, we excluded three copies of data with a history of mental illness and medication in the past three years. Most participants were right-handed (∼96%). Nine participants reported their meditation practice experiences in the past three years. In the pretest, we included data from 67 participants. In the posttests, we included data from 58 participants after excluding five participants who specified the wrong meditation type. The level of meditation involvement did not differ significantly between intertemporal (*M *= 70.74, *SD *= 20.20) and mindfulness meditations (*M *= 63.42, *SD *= 25.00). (We reported the effect size (Cohen’s *d* = 0.32) for further interpretation because the *P*-value was close to the threshold of statistical significance (*P* = .053).)

**Figure 3.**
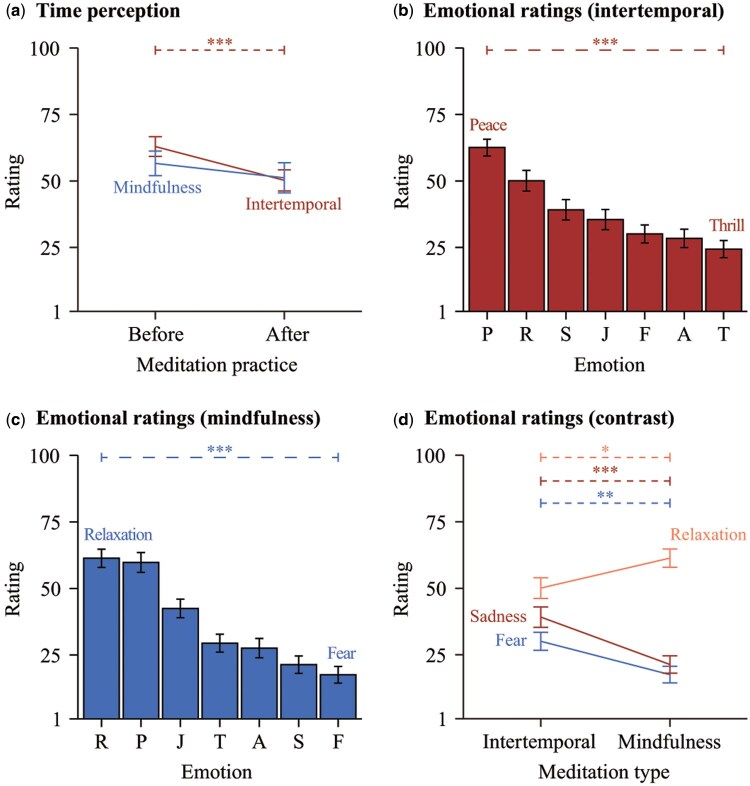
Behavioural effects of mindfulness and intertemporal meditations. Each panel displays error bars (*M* ± *SE*) and the level of significance (^*^P < .05, ^**^P < .01, ^***^P < .001). Panel (a) displays time perception variations before and after intertemporal and mindfulness meditation practices, as indicated by the corresponding labels in the figure. Panels (b) and (c) display the differences in self-reported emotional experience (emotional ratings) after the intertemporal meditation and the mindfulness meditation, respectively. Panel (d) displays the rating differences for seven emotions, each indicated by its label, between two meditation conditions. P = Peace, R = Relaxation, S = Sadness, J = Joy, F = Fear, A = Anxiety, T = Thrill.

*Time perception.* Participants, after the practice of intertemporal meditation, accelerated their perception of time more than before (see Fig. 3a), *t* (34) = 4.05, *P* < .001, Cohen’s *d *= 0.56. However, time perception did not significantly change before and after mindfulness meditation, *t* (27) = 1.83, *P* = .078. Time perception did not reveal any differences between the two types of meditation practices, *t* (50) = 0.14, *P* = .891. To further examine whether it was the second meditation type specifically modulated time perception or it was a combined effect, we compared the changes in time perception (posttest minus pretest) between two meditation types. Although the intertemporal meditation group showed a reduction in perceived time length (*M *=* −*12.71, *SD *= 18.59), compared to the mindfulness meditation group (*M *=* −*5.43, *SD *= 15.69), this difference did not reach statistical significance, *t* (61) = 1.69, *P* = .097. This result suggests a combined effect of mindfulness versus intertemporal meditation.

*Self-reported emotional experience ratings.* As shown in Fig. 3b, the participants after the practice of intertemporal meditation gave the emotional experience of *peace* the highest rating (*M *= 62.55, *SD *= 22.89) and *thrill* the lowest rating (*M *= 24.36, *SD *= 23.50), *F* (6, 394) = 18.11, *P* < .001, partial *η^2^* = 0.22. In contrast, the participants after the practice of mindfulness meditation gave their highest emotional rating to the experience of *relaxation* (*M *= 61.33, *SD *= 24.51) and the lowest rating to *fear* (*M *= 17.53, *SD *= 22.25) (see Fig. 3c), *F* (6, 395) = 28.39, *P* < .001, partial *η^2^* = 0.30. Revealed by the paired post-hoc comparisons, the ratings of *peace* and *relaxation* after the practices of both meditations were significantly higher than other emotions, *P*s < .001 [Table S.3 (see online supplementary material for a colour version of this table) provides detailed results of Tukey HSD post-hoc analysis].

By comparing the self-reported emotional experience ratings across the two meditation conditions (see Fig. 3d), participants after the practice of intertemporal meditation demonstrated a combination of higher levels of *fear*, *t* (99) = 2.73, *P* = .008, Cohen’s *d *= 0.54; and *sadness*, *t* (100) = 3.54, *P* = .001, Cohen’s *d *= 0.69. After the practice of mindfulness meditation, the participants demonstrated a higher level of *relaxation*, *t* (98) = 2.16, *P* = .033, Cohen’s *d *= 0.43.

To address potential gender differences in emotional response, we conducted linear regression analyses predicting each emotional rating from meditation type, gender, and their interaction (see Table 1 for more details). The results revealed a significant main effect of gender on *thrill*, with male participants reporting higher ratings across conditions (*β*  =  15.78, *P* = .012). A significant main effect of meditation type was found for *relaxation* (*β* = *−*12.85, *P* = .037), with participants reporting lower ratings after intertemporal meditation than after mindfulness. For *sadness*, participants after intertemporal meditation reported higher scores than those after mindfulness (*β*  =  15.59, *P* = .012). No significant interaction effects between gender and meditation type were observed for any emotion.

*Time perception changes in predicting emotional experience ratings.* We conducted a multiple regression analysis to examine whether the relationship between time perception change (posttest minus pretest) and emotional experience differed across meditation types (see Table 2 for more details). Among the seven emotions, only *sadness* showed a significant interaction effect. Specifically, there was a significant main effect of time perception change (*β* = *−*0.74, *P* = .035), a main effect of meditation type (*β*  =  25.07, *P* = .003), and a significant interaction between the two (*β*  =  1.11, *P* = .012). This suggests that time perception change predicted greater sadness, and this relationship was stronger in the intertemporal meditation condition. The full model explained 19.3% of the variance in sadness ratings (*R*^2^ = 0.193, *F* (3, 52) = 4.13, *P* = .011).

Additionally, meditation type had significant main effects on *joy* (*β* = *−*18.35, *P* = .018), with the self-reported emotional experience ratings being lower after intertemporal meditation compared to mindfulness. The full model explained 14.9% of the variance in joy ratings (*R^2^* = .149, *F* (3, 52) = 3.03, *P* = .038).

### Hemodynamic response

*Preprocessed hemodynamic data.* We included hemodynamic data from 52 participants (23 males and 29 females). We excluded three participants with a history of mental illness or medication history in the past three years. After preprocessing, we pruned an average of 31% of the channels and preserved the data from 25 channels. These channels covered the following brain regions: 18 in the prefrontal lobe (channels 1–9, 11–12, and 14–20), three in the left temporal lobe (channels 22, 26, and 29), two in the right temporal lobe (channels 24 and 32), and two in the occipital lobe (channel 35 and 36). These channels covered eight brain regions including BA9, BA10, BA11, BA45, BA46, BA2, BA21, and BA17.

To assess potential global differences across brain regions (channels), we conducted repeated-measures MANOVA on the averaged HbO responses across all activated channels for each session. None of the three sessions showed significant multivariate effects between meditation types (Session 1: *P* = .939; Session 2: *P* = .394; Session 3: *P* = .924), suggesting no widespread spatial shift in activation, and the effects were likely localized.

To further examine the spatial structure of the fNIRS data, we also computed inter-channel Pearson correlations within each meditation condition. Across all sessions, the correlations were moderate (mean *r* ranging from 0.22 to 0.30), with similar magnitudes for mindfulness (Session 1: $\bar{r}$ = 0.26, *SD *= 0.29; Session 2: $\bar{r}$ = 0.22, *SD *= 0.29; Session 3: $\bar{r}$ = 0.27, *SD *= 0.23) and intertemporal meditation (Session 1: $\bar{r}$ = 0.27, *SD *= 0.25; Session 2: $\bar{r}$ = 0.30, *SD *= 0.30; Session 3: $\bar{r}$ = 0.28, *SD *= 0.29).

To further test localized effects between the two meditation conditions, we used channel-wise analyses, a standard practice in within-subject fNIRS research, where strong spatial independence assumptions do not hold (Pinti et al. 2020).

To identify meaningful activation differences, we adopted a conservative dual-criterion approach (*P* < .001 and Cohen’s *d *> 0.5), emphasizing robust regional effect estimates over assumptions of global spatial reorganization (Chen et al. 2017). A channel was considered activated by meditation if both criteria were met. Channel activation differences between the two meditation types were revealed by paired *t*-tests across meditation sessions. Hemispheric activation differences between the two meditation types were revealed in BA9, BA10, BA11, BA46, BA45, BA21, and BA17. We reported the one-sample *t*-test results for the activation detection of each channel during intertemporal and mindfulness meditation in Tables S.4 and S.5 (see online supplementary material for a colour version of these tables).

**Figure 4.**
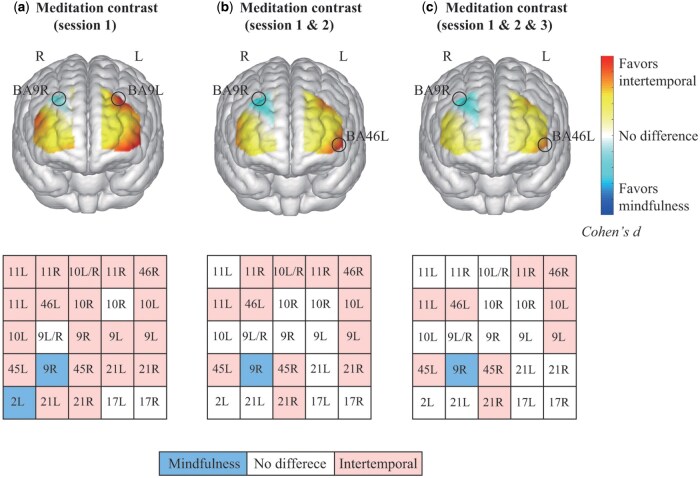
Channel activation contrast between mindfulness and intertemporal meditations. Panels (a), (b), and (c) display meditation contrast (i.e. intertemporal—mindfulness) of hemodynamic activations for all valid channels during the first session, the first two sessions, and the overall practice, respectively. The graphs were presented using the EasyTopo toolbox (Tian et al. 2012) in MATLAB. Each panel displays the activation contrast in the prefrontal lobe and corresponding Brodmann areas with the most significant effects, as indicated by its legend. Additionally, the table in each panel denotes the relatively higher activation during the intertemporal meditation (cells marked "Intertemporal"), the relatively higher activation during the mindfulness meditation (cells marked "Mindfulness"), and no difference between two meditations (cells marked "No difference").

*Channel activation contrast.* During the first session (see Fig. 4a), intertemporal meditation elicited significantly higher bilateral activations than mindfulness meditation in 19 channels (*P*s < .001, average Cohen’s *d *= 1.43) across BA10, BA11, BA45, BA46, and BA21. In sharp contrast, mindfulness meditation elicited higher bilateral activations in only two channels across BA9 and BA2 (*P*s < .001, average Cohen’s *d *= 0.95). The largest activation differences were found in BA9, where intertemporal meditation induced higher activation in the left hemisphere of BA9 (channel 17), Cohen’s *d *= 2.60, and mindfulness meditation induced higher activation in the right hemisphere of BA9 (channel 19), Cohen’s *d *= 0.96.

After combining the first and second sessions (see Fig. 4b), intertemporal meditation showed higher activations in 12 channels (*P*s < .001, average Cohen’s *d *= 1.06) in BA9, BA10, BA11, BA45, BA46, and BA21. The largest difference was observed in the left hemisphere of BA46 (channel 7) during intertemporal meditation, Cohen’s *d *= 1.76. Mindfulness meditation, on the other hand, showed higher activation in one channel (*P* < .001, Cohen’s *d *= 1.02) in the right hemisphere of BA9 (channel 19).

Across the entire meditation practice (see Fig. 4c), intertemporal meditation resulted in higher activations in 9 channels (*P*s < .001, average Cohen’s *d *= 0.92) covering BA9, BA10, BA11, BA45, BA46, and BA21. The left hemisphere of BA46 (channel 7) again showed the largest difference with intertemporal meditation, Cohen’s *d *= 1.38. Mindfulness meditation elicited higher activation in one channel (*P* < .001, Cohen’s *d *= 0.93) in the right hemisphere of BA9 (channel 19). See Table S.6 (see online supplementary material for a colour version of this table) for more details.

**Figure 5.**
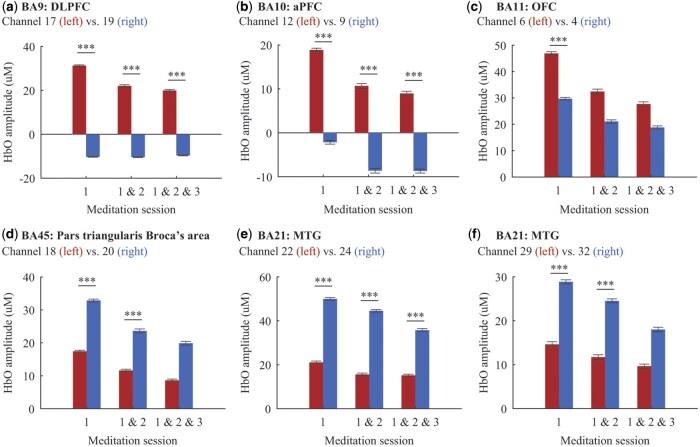
Hemispheric activation contrast between mindfulness and intertemporal meditations. The graphs display the meditation contrast (i.e. intertemporal—mindfulness) of hemodynamic activations in the regions of BA9 (panel a), BA10 (panel b), BA11 (panel c), BA45 (panel d), and BA21 (panels e and f). Each panel denotes the left hemisphere activation in red bars and the right hemisphere activation in blue bars (*M* ± *SE*). ^***^P < .001.

*Hemispheric activation contrast.* Intertemporal meditation elicited more activations in the left BA9 (see Fig. 5a), whereas mindfulness meditation elicited more in the right hemisphere (session 1: Cohen’s *d *= 4.00; session 1 & 2: Cohen’s *d *= 1.84; session 1 & 2 & 3: Cohen’s *d *= 1.71).

In BA10 (see Fig. 5b), intertemporal meditation showed higher activation in the left hemisphere, while mindfulness meditation showed more in the right hemisphere (session 1: Cohen’s *d *= 1.26; session 1 & 2: Cohen’s *d *= 0.83; session 1 & 2 & 3: Cohen’s *d *= 0.80). In BA11 (see Fig. 5c), intertemporal meditation elicited higher activations in the left hemisphere during the first session (Cohen’s *d *= 0.77).

In BA45 (see Fig. 5d), intertemporal meditation showed greater activations in the right hemisphere during the first session (Cohen’s *d *= 1.07) and the first two sessions (Cohen’s *d *= 0.55). For BA21 (see Fig. 5e and f), intertemporal meditation elicited higher activations in the right hemisphere across all sessions (session 1: average Cohen’s *d *= 0.91; session 1 & 2: average Cohen’s *d *= 0.85; session 1 & 2 & 3: Cohen’s *d *= 0.71).

*Additional hemodynamic results in meditation-experienced participants.* Given that prior meditation experience may modulate how individuals engage with meditation practices, we conducted an additional hemodynamic analysis specifically on participants who reported having prior meditation experiences. Although the sample size was limited (*n *= 7, including 5 females), this analysis offers preliminary insight into baseline modulation effects.

Across 24 valid channels, intertemporal meditation elicited stronger activations than mindfulness meditation in 21 channels after the first session (*P*s < .001, average Cohen’s *d *= 3.83), in 21 channels after the first and second sessions combined (*P*s < .001, average Cohen’s *d *= 2.47), and in 20 channels after all three sessions (*P*s < .001, average Cohen’s *d *= 1.87). By contrast, mindfulness meditation consistently produced stronger activations in the right BA9 (channel 15) (session 1: Cohen’s *d *= 2.23; session 1 & 2: Cohen’s *d *= 1.88; session 1 & 2 & 3: Cohen’s *d *= 1.52). See Table S.7 (see online supplementary material for a colour version of this table) for more statistical details.

## Discussion

The present study provided experimental evidence to verify the potential effectiveness of intertemporal meditation in regulating time perception and emotional experiences for young adults. Behavioural results showed that participants exhibited a significantly accelerated perception of time following intertemporal meditation but not after mindfulness meditation, partially supporting *Hypothesis 1*. Given that time perception was only measured twice (before and after two meditations), and the between-meditation difference did not reach significance (*P* = .097), an alternative explanation for the significant difference in time perception may be better understood as a contrast effect between mindfulness versus intertemporal meditation. That is, mindfulness followed by intertemporal meditation resulted in a faster time perception than the opposite sequence.

As shown in Fig. 3, the participants reported higher levels of *peace* and *relaxation* and lower levels of *fear* and *anxiety* following intertemporal meditation. Although intertemporal meditation elicited higher ratings of *fear* and *sadness* than mindfulness meditation, their average ratings remained low, indicating minimal impact. These findings supported *Hypothesis 2*, suggesting that intertemporal meditation induces adaptive emotional responses (more peace and relaxation) rather than defensive ones (more fear and anxiety).

Additional analyses examined whether these effects were moderated by gender. The results revealed that male participants reported significantly higher *thrill* ratings than female participants regardless of meditation type. However, no significant gender plus meditation-type interaction effects were observed, suggesting that the emotional benefits of intertemporal meditation—particularly the increases in peace and relaxation—were largely consistent across genders.

Partially supporting *Hypothesis 3*, changes in time perception were significantly associated with emotional experiences following intertemporal meditation. Specifically, a greater acceleration in time perception predicted lower levels of *sadness*, and this relationship was significantly stronger after intertemporal meditation than after mindfulness meditation. These findings suggest that intertemporal meditation may adaptively regulate emotional responses, particularly sadness, rather than fear or anxiety, by reshaping subjective time through episodic future thinking.

Consistent with the behavioural results, the hemodynamic measures indicated activations in several key brain regions related to emotion regulation and cognitive control. In contrast to the right-lateralized activations due to mindfulness meditation, intertemporal meditation revealed a pattern of left-lateralized activations in BA9 (DLPFC) and BA10 (aPFC). The left hemisphere is implicated in the approach-related emotions, typically positive, which motivate engagement and goal pursuit. In contrast, the right hemisphere specializes in withdrawal-related emotions, typically negative, prompting avoidance and retreat (e.g. Irwin et al. 2004, Versace et al. 2010, Ross 2021). A greater left activation due to intertemporal meditation suggests an adaptive regulation of emotional experience.

Both aPFC (Koch et al. 2018, Kaldewaij et al. 2021) and DLPFC (Kanske et al. 2011, Martins et al. 2015) play a crucial role in self-control capacities and emotion regulation. Studies using transcranial direct current stimulation have shown that enhancing cortical excitability in the left DLPFC can modulate emotional fluctuations and alleviate negative emotions (Allaert et al. 2019, Nejati et al. 2021). Similarly, repetitive transcranial magnetic stimulation to the left DLPFC has been found to significantly improve emotion regulation and facilitate weight gain in anorexia nervosa patients (Dalton et al. 2021). By contrast, enhancing excitability in the right DLPFC has been associated with emotional dysfunction and negative emotional outcomes (Sanchez-Lopez et al. 2018). The aPFC is involved in emotional control by adaptively adjusting emotional strategies to meet personal or situational demands (Koch et al. 2018, Kaldewaij et al. 2021). A meta-analysis indicated that the left hemisphere of the aPFC exhibits higher activations during cognitive reappraisal, underscoring its importance in emotional strategy evaluation (Morawetz et al. 2017). The complementary roles of the DLPFC and aPFC in regulating emotions and cognitive control highlight intervention potentials of mixed exercises of mindfulness and intertemporal meditation in enhancing cognitive control and emotion regulation.

Furthermore, intertemporal meditation elicited higher activations in the OFC compared to mindfulness meditation and stronger left-hemisphere activations during the initial session. The OFC is crucial for evaluating emotional stimuli and relaying information to brain areas like the DLPFC (Bechara et al. 2000, Kringelbach and Rolls 2004, Rolls 2019). OFC activations are also linked with cognitive reappraisal strategies, which help down-regulate subjective emotional experiences by reducing amygdala excitability (Kalisch 2009, Kanske et al. 2011). Cognitive reappraisal is an adaptive strategy that involves reinterpreting the meaning of emotional responses to manage emotional fluctuations (Gross 1998, Augustine and Hemenover 2009). Studies have shown significantly stronger left OFC activations over the right side when processing negative emotional faces (Altshuler et al. 2008, Versace et al. 2010). This activation pattern suggests that mental travels between the present and the end of life may elicit cognitive reappraisal in young adults, enhancing their ability to regulate emotional experiences induced by episodic future thinking throughout their lifespan.

The hemodynamic measures also revealed a pattern of right-lateralized activations in BA45 (Pars triangularis Broca’s area) and BA21 (MTG) during intertemporal meditation. Broca’s area, particularly BA45, is traditionally associated with language production and processing, primarily in the left hemisphere (Heim et al. 2005). However, right hemisphere activation in this region might indicate the interpretation of social cues and emotional tones during language processing (Wildgruber et al. 2005, Jabbi and Keysers 2008). In meditation practices, higher activations in the right Broca’s area might indicate enhanced abilities in processing emotional and social nuances during intertemporal meditation. Similarly, the right-lateralized activation in MTG is traditionally associated with episodic memory and mental time travel (Botzung et al. 2008, Viard et al. 2011). During intertemporal meditation, heightened activation in the right MTG might reflect enhanced engagement in processes related to autobiographical memory retrieval and episodic future thinking from the present to the end of life (Spreng and Grady 2010). Our participants seemed to be more engaged in intertemporal meditation than mindfulness meditation (*P* = .053, Cohen’s *d *= 0.32). This pattern was further supported by our exploratory analysis, which showed that participants with prior meditation experience exhibited stronger overall brain activation during intertemporal meditation compared to mindfulness. It appears that individuals with prior meditation experience are better able to discern the differences between the two types of meditation. This finding further supports the distinctiveness of the underlying neural mechanisms associated with mindfulness meditation and intertemporal meditation.

These above findings suggest that young adults were not merely ruminating on mortality but actively integrating this contemplation into their personal narrative, fostering a recontextualization of emotional experiences in light of life’s finitude.

Previous research on mortality salience has primarily emphasized the moment of death, often triggering fear and anxiety (Mroczek and Almeida 2004, Navarrete and Fessler 2005, Boyer 2007). In contrast, the present study introduces the adaptive time management as a novel framework that departs from the defensive emphasis of TMT, emphasizing individuals’ capacity to actively regulate temporal priorities according to life stages and situational demands (Wang et al. 2019, Lu et al. 2023, Wang and Wang 2024). For young adults, intertemporal meditation may shift time perspective and encourage the prioritization of present emotional needs—an adaptive strategy for coping with existential anxiety and motivational uncertainty (Thomas et al. 2016).

Beyond the theoretical contributions, this study also highlights the potential of intertemporal meditation as an experiential intervention tool in applied settings. By encouraging individuals to simulate the temporal trajectory towards the end of life, this practice may promote not only emotional acceptance but also a more integrated sense of purpose and time. In clinical contexts such as palliative care, grief-focused psychotherapy, or existential therapy, such guided mental time travel could help patients cultivate a calm and meaningful relationship with mortality—supporting emotion regulation, alleviating death anxiety, and enhancing psychological resilience (Yalom 2020, Xunlin et al. 2020). Moreover, integrating this approach into death education curricula may enrich experiential learning by enabling participants to engage vividly with their own imagined futures. This could foster the development of more personal and nuanced understandings of life priorities, legacy, and the concept of a ‘good death’, potentially improving both mental well-being and adaptive decision-making.

However, our exploratory research has its limitations. First, the accumulated hemodynamic responses during both meditations decreased gradually as the meditation practice went through. This may be due to the setup of the different durations over these practice sessions. Further study should aim to make sure each meditation session has an equal duration. Second, given the current design, we were unable to directly compare the emotional changes before and after each type of meditation. Future studies may consider a between-subject design for the two types of meditation. Third, our present study did not explore the relationship between accelerated time perception and enhanced cognitive function following intertemporal meditation. Understanding this relationship is crucial for revealing the mechanisms of intertemporal meditation and developing targeted cognitive interventions. Further studies may implement longitudinal designs and more detailed neuroimaging techniques (e.g. fMRI) to investigate the dynamic relationship between time perception changes and cognitive function improvements.

## Conclusion

As a result of the most recent development in our studies, we tested the behavioural and neural effects of a novel practice of intertemporal meditation. Intertemporal meditation accelerates time perception, whereas mindfulness meditation does not. Descriptively, the intertemporal meditation resulted in an emotional state of feeling peaceful with a mix of slight sadness, joy, and fear. In contrast, the mindfulness meditation induced an emotional state that was relaxed with some joy. Hemodynamic measures during intertemporal meditations revealed significant left-lateralized activations in brain regions associated with emotion regulation and cognitive control, particularly the DLPFC, aPFC, and OFC. Right-lateralized activations in Broca’s area and MTG indicated enhanced social cues and autobiographical memory processing. These findings underscore the adaptive utility of intertemporal meditation in emotion regulation and highlight its potential for strengthening cognitive control functions.

## Supplementary Material

nsaf080_Supplementary_Data

## Data Availability

Besides the supplement material, all data, code, and figures are available at an online repository (https://github.com/Cellphonexf/Intertemporal-Meditation-fNIRS.git). Additional request should be made to the corresponding author.
