# Supplementary material for: Intertemporal meditation regulates time perception and emotions: an exploratory fNIRS study
Source: Soc Cogn Affect Neurosci. 2025 Aug 1;20(1):nsaf080. doi: 10.1093/scan/nsaf080 (PMC12399512; doi:10.1093/scan/nsaf080)
Supplement: nsaf080_Supplementary_Data [file nsaf080_supplementary_data.zip › scan-24-189-76-83.pdf]

Supplement Material

Table S.1 Instructions for meditation practices

Table S.2 fNIRS channel coordinates and corresponding Brodmann areas

Table S.3 Post-hoc comparisons on self-reported emotional experience

Table S.4 Hemodynamic activation detection for intertemporal meditation

Table S.5 Hemodynamic activation detection for mindfulness meditation

Table S.6 Channel activation contrast between two meditations

Table S.7 Channel activation contrast between two meditations in experienced participants

For Peer Review

Table S.1 Instructions for Meditation Practices

Intertemporal meditation	Mindfulness meditation
**Session 1 (300 Seconds)**	
Please maintain your posture and focus on your breathing. Follow my rhythm: inhale, exhale, inhale, exhale. Feel the air slowly flow into your nose, mouth, and lungs, then exhale it out. Let the thoughts and ideas in your mind leave with your breath. Inhale, exhale.<br>Imagine your life passage as a time tunnel. You walk alone along it towards a distant destination as time passes. Step by step, synchronized with the rhythm of your breath, **you get closer to the end.** Inhale, exhale.<br>Now, pause for a moment **and focus on the end of life.** What is the scene of life here? Is it bright or dark, warm or cold? **Use your senses to feel the end of life;** what do you see, hear, smell? Feel and experience **the end time of life.** Inhale, exhale.<br>Think about what you have strived for your whole life—your studies, work, money, and honors.<br>Consider the time and effort you have put into these and what they meant to you. Think about your family, friends, and your significant other. Imagine them by your side at this moment; what does it feel like?<br>Focus on this moment and feel your feelings. Inhale, exhale.<br>**The past is gone, and the future will not come. Let go of the past and now, focusing on this end moment in life. Silently repeat after me: I am about to leave at this moment, I'll be gone, I'll be gone, I'll be gone at this place.**<br>Now, I will count to three. You can then slowly open your eyes. Three, two, one (and a bell sound).	Please maintain your posture and focus on your breathing. Follow my rhythm: inhale, exhale, inhale, exhale. Feel the air slowly flow into your nose, mouth, and lungs, then exhale it out. Let the thoughts and ideas in your mind leave with your breath. Inhale, exhale.<br>Imagine your life passage as a time tunnel. You walk alone along it towards a distant destination as time passes. Step by step, synchronized with the rhythm of your breath, **you are moving ahead.** Inhale, exhale.<br>Now, pause for a moment and **focus on the present moment in life.** What is the scene of life here? Is it bright or dark, warm or cold? **Use your senses to feel this moment;** what do you see, hear, smell? Feel and experience **the present moment.** Inhale, exhale.<br>Think about what you have strived for until now—your studies, work, money, and honors.<br>Consider the time and effort you have put into these and what they meant to you. Think about your family, friends, and your significant other. Imagine them by your side at this moment; what does it feel like?<br>Focus on this moment and feel your feelings. Inhale, exhale.<br>**The past is gone, and the future has not yet arrived. Let go of the past and future, focusing on this present moment. Silently repeat after me: I live in this moment, I live in this moment, I live in this place.**<br>Now, I will count to three. You can then slowly open your eyes. Three, two, one (and a bell sound).
**Session 2 (60 Seconds)**	
Please maintain your breathing rhythm: inhale, exhale, inhale, exhale. **Return to the scene of the end of life.** Is it bright or dark here? Warm or cold? Noisy or quiet? **Engage all your senses to feel the end moment of life.** Inhale, exhale. Three, two, one (and a bell sound).	Please maintain your breathing rhythm: inhale, exhale, inhale, exhale. **Return to the scene of our current moment.** Is it bright or dark here? Warm or cold? Noisy or quiet? **Engage all your senses to feel this moment.** Inhale, exhale. Three, two, one (and a bell sound).
**Session 3 (60 Seconds)**	
Same as the instruction of session 2.	Same as the instruction of session 2.

Note. The recorded instructions for intertemporal meditation are copyright-protected, and a written request for consent to reuse the materials from the corresponding author is required.

For Peer Review

Table S.2 The fNIRS Channel Coordinates and Corresponding Brodmann Areas

ROI	Channel	10-10 system (source-detector)	MNI coordinate (*X*, *Y*, *Z*)	Brodmann area: anatomy	Anatomical overlap probability (%)
Prefrontal lobe	1	Fpz-Fp1	(-11, 73, -5)	11L: Orbitofrontal cortex	56%
	2	Fpz-Fp2	(14, 73, -5)	11R: Orbitofrontal cortex	61%
	3	Fpz-AFz	(3, 69, 14)	10L/R: Anterior prefrontal cortex	100%
	4	AF8-Fp2	(37, 65, -10)	11R: Orbitofrontal cortex	43%
	5	AF8-F6	(52, 48, 0)	46R: Dorsolateral prefrontal cortex	79%
	6	AF7-Fp1	(-34, 64, -9)	11L: Orbitofrontal cortex	47%
	7	AF7-F5	(-49, 49, -1)	46L: Dorsolateral prefrontal cortex	87%
	8	AF4-Fp2	(29, 69, 5)	10R: Anterior prefrontal cortex	69%
	9	AF4-AFz	(17, 68, 25)	10R: Anterior prefrontal cortex	98%
	10	AF4-F6	(45, 55, 16)	46R: Dorsolateral prefrontal cortex	85%
	11	AF3-Fp1	(-26, 68, 4)	10L: Anterior prefrontal cortex	64%
	12	AF3-AFz	(-14, 68, 24)	10L: Anterior prefrontal cortex	99%
	13	AF3-F5	(-42, 55, 15)	46L: Dorsolateral prefrontal cortex	84%
	14	Fz-AFz	(1, 55, 41)	9L/R: Dorsolateral prefrontal cortex	93%
	15	Fz-F2	(13, 46, 53)	9R: Dorsolateral prefrontal cortex	74%
	16	Fz-F1	(-10, 46, 52)	9L: Dorsolateral prefrontal cortex	76%
	17	F3-F1	(-30, 45, 41)	9L: Dorsolateral prefrontal cortex	79%
	18	F3-F5	(-47, 43, 24)	45L: Pars triangularis Broca's area	83%
	19	F4-F2	(34, 45, 43)	9R: Dorsolateral prefrontal cortex	86%
	20	F4-F6	(50, 43, 26)	45R: Pars triangularis Broca's area	89%
Temporal lobe	21	FT7-FT9	(-61, 2, -30)	21L: Middle temporal gyrus	84%
	22	FT7-T7	(-69, -8, -14)	21L: Middle temporal gyrus	100%
	25	C5-T7	(-70, -20, 6)	22L: Superior temporal gyrus	68%
	26	C5-CP5	(-69, -34, 27)	2L: Primary somatosensory cortex	34%
	29	TP7-T7	(-70, -36, -11)	21L: Middle temporal gyrus	51%
	30	TP7-CP5	(-69, -48, 9)	22L: Superior temporal gyrus	49%
	31	TP7-TP9	(-63, -50, -23)	20L: Inferior temporal gyrus	53%
	23	FT8-FT10	(63, 2, -29)	21R: Middle temporal gyrus	84%
	24	FT8-T8	(70, -8, -12)	21R: Middle temporal gyrus	100%
	27	C6-T8	(73, -21, 8)	22R: Superior temporal gyrus	77%
	28	C6-CP6	(70, -35, 29)	40R: Supramarginal gyrus part of Wernicke's area	41%
	32	TP8-T8	(72, -37, -10)	21R: Middle temporal gyrus	52%
	33	TP8-CP6	(69, -50, 11)	22R: Superior temporal gyrus	50%
	34	TP8-TP10	(64, -52, -22)	37R: Fusiform gyrus	62%
Occipital lobe	35	O1-Oz	(-14, -107, 10)	17L: Primary visual cortex	100%
	36	O2-Oz	(14, -106, 10)	17R: Primary visual cortex	100%

Note. Only the Brodmann area with the largest overlap probabilities were listed. L = left, R = right.

For Peer Review

Table S.3 Post-hoc Comparisons for Self-reported Emotional Experience

Meditation type	Comparison	Mean difference	95% CI	Adjusted *p*-value
Intertemporal	Peace > Anxiety	36.40	[22.29, 50.51]	<. 001
	Peace > Fear	35.47	[21.23, 49.71]	< .001
	Peace > Joy	27.57	[13.52, 41.62]	< .001
	Peace > Sadness	27.03	[12.92, 41.14]	< .001
	Peace > Thrill	39.55	[25.50, 53.60]	< .001
	Relaxation > Anxiety	23.36	[9.25, 37.47]	<. 001
	Relaxation > Fear	22.43	[8.20, 36.67]	< .001
	Relaxation > Joy	14.53	[0.49, 28.58]	= .037
	Relaxation > Thrill	26.52	[12.47, 40.57]	< .001
Mindfulness	Relaxation > Anxiety	31.46	[17.71, 45.21]	< .001
	Relaxation > Fear	42.08	[29.39, 55.77]	< .001
	Relaxation > Joy	17.72	[3.97, 31.47]	= .003
	Relaxation > Sadness	39.08	[25.39, 52.77]	< .001
	Relaxation > Thrill	31.21	[17.52, 44.90]	< .001
	Peace > Anxiety	30.54	[16.91, 44.17]	< .001
	Peace > Fear	41.16	[27.59, 54.72]	< .001
	Peace > Joy	16.80	[3.17, 30.43]	= .005
	Peace > Sadness	38.16	[24.59, 51.72]	< .001
	Peace > Thrill	30.29	[16.72, 43.86]	< .001
	Joy > Fear	24.35	[10.73, 37.98]	< .001
	Joy > Sadness	21.35	[7.73, 34.98]	< .001
	Joy > Anxiety	13.74	[0.05, 27.42]	= .049

Note. Mean differences were reported with the second emotion in each pair serving as the reference. Confidence intervals (CIs) reflected the 95% probability that the true mean difference lied within the interval. *P*-values were adjusted for multiple comparisons via Tukey HSD, with significant results highlighted.

For Peer Review

Table S.4 Hemodynamic Activation Detection for Intertemporal Meditation

		Session 1 (n = 1526)		Session 1 & 2 (n = 1832)		Session 1 & 2 & 3 (n = 2138)	
Channel	Brodmann area	t-value (p)	*Cohen's d*	t-value (p)	*Cohen's d*	t-value (p)	*Cohen's d*
1	11L	110.97 (< .001)	**2.84***	61.50 (< .001)	**1.44***	54.88 (< .001)	**1.19***
2	11R	112.33 (< .001)	**2.88***	63.11 (< .001)	**1.47***	54.40 (< .001)	**1.18***
3	10L/R	81.70 (< .001)	**2.09***	52.58 (< .001)	**1.23***	50.30 (< .001)	**1.09***
4	11R	76.87 (< .001)	**1.97***	53.43 (< .001)	**1.25***	45.57 (< .001)	**0.99***
5	46R	47.47 (< .001)	**1.22***	40.13 (< .001)	**0.94***	39.03 (< .001)	**0.84***
6	11L	73.03 (< .001)	**1.87***	57.91 (< .001)	**1.35***	46.04 (< .001)	**1.00***
7	46L	33.80 (< .001)	**0.87***	40.33 (< .001)	**0.94***	36.49 (< .001)	**0.79***
8	10R	78.37 (< .001)	**2.01***	41.63 (< .001)	**0.97***	35.40 (< .001)	**0.77***
9	10R	49.78 (< .001)	**1.27***	32.83 (< .001)	**0.77***	32.22 (< .001)	**0.70***
11	10L	77.45 (< .001)	**1.98***	47.75 (< .001)	**1.12***	47.80 (< .001)	**1.03***
12	10L	73.25 (< .001)	**1.88***	59.28 (< .001)	**1.39***	59.93 (< .001)	**1.30***
14	9L/R	38.66 (< .001)	**0.99***	31.64 (< .001)	**0.74***	33.36 (< .001)	**0.72***
15	9R	80.46 (< .001)	**2.06***	70.37 (< .001)	**1.64***	54.46 (< .001)	**1.18***
16	9L	49.73 (< .001)	**1.27***	46.56 (< .001)	**1.09***	39.58 (< .001)	**0.86***
17	9L	125.70 (< .001)	**3.22***	95.11 (< .001)	**2.22***	87.62 (< .001)	**1.89***
18	45L	85.41 (< .001)	**2.19***	62.44 (< .001)	**1.46***	51.81 (< .001)	**1.12***
19	9R	36.38 (< .001)	**0.93***	38.20 (< .001)	**0.89***	37.82 (< .001)	**0.82***
20	45R	108.82 (< .001)	**2.79***	69.67 (< .001)	**1.63***	59.61 (< .001)	**1.29***
22	21L	-19.16 (< .001)	NA	-7.20 (< .001)	NA	-3.76 (< .001)	NA
24	21R	15.12 (< .001)	NA	23.56 (< .001)	**0.55***	22.63 (< .001)	NA
26	2L	-82.51 (< .001)	NA	-32.74 (< .001)	NA	-29.31 (< .001)	NA
29	21L	19.37 (< .001)	NA	27.19 (< .001)	**0.64***	28.64 (< .001)	**0.62***
32	21R	37.14 (< .001)	**0.95***	41.65 (< .001)	**0.97***	32.83 (< .001)	**0.71***
35	17L	-47.79 (< .001)	NA	-48.77 (< .001)	NA	-51.91 (< .001)	NA
36	17R	-6.67 (< .001)	NA	-17.63 (< .001)	NA	-23.69 (< .001)	NA

Note. One-sample t-tests were used to detect the hemodynamic activations during the intertemporal meditation. * denotes that the channel was activated (i.e., t-value > 0, p < .001, *Cohen's d* > 0.5). NA indicates that the channel was not activated during meditation. L = left, R = right.

For Peer Review

Table S.5 Hemodynamic Activation Detection for Mindfulness Meditation

		Session 1 (n = 1526)		Session 1 & 2 (n = 1832)		Session 1 & 2 & 3 (n = 2138)	
Channel	Brodmann area	*t*-value (*p*)	*Cohen's d*	*t*-value (*p*)	*Cohen's d*	*t*-value (*p*)	*Cohen's d*
1	11L	54.22 (< .001)	**1.39***	66.26 (< .001)	**1.55***	62.77 (< .001)	**1.36***
2	11R	65.97 (< .001)	**1.69***	75.02 (< .001)	**1.75***	69.68 (< .001)	**1.51***
3	10L/R	73.35 (< .001)	**1.88***	86.73 (< .001)	**2.03***	82.03 (< .001)	**1.77***
4	11R	21.05 (< .001)	**0.54***	26.55 (< .001)	**0.62***	23.61 (< .001)	**0.51***
5	46R	-7.57 (< .001)	NA	-5.03 (< .001)	NA	-0.63 (= .528)	NA
6	11L	-21.78 (< .001)	NA	-0.98 (= .328)	NA	-2.04 (= .041)	NA
7	46L	-67.25 (< .001)	NA	-36.97 (< .001)	NA	-29.62 (< .001)	NA
8	10R	63.96 (< .001)	**1.64***	65.42 (< .001)	**1.53***	54.62 (< .001)	**1.18***
9	10R	76.98 (< .001)	**1.97***	74.22 (< .001)	**1.73***	73.06 (< .001)	**1.58***
11	10L	39.71 (< .001)	**1.02***	47.80 (< .001)	**1.12***	48.13 (< .001)	**1.04***
12	10L	66.87 (< .001)	**1.71***	76.36 (< .001)	**1.78***	80.28 (< .001)	**1.74***
14	9L/R	52.96 (< .001)	**1.36***	59.75 (< .001)	**1.40***	64.35 (< .001)	**1.39***
15	9R	98.15 (< .001)	**2.51***	107.29 (< .001)	**2.51***	90.76 (< .001)	**1.96***
16	9L	25.70 (< .001)	**0.66***	34.04 (< .001)	**0.80***	41.72 (< .001)	**0.90***
17	9L	72.27 (< .001)	**1.85***	67.84 (< .001)	**1.59***	67.07 (< .001)	**1.45***
18	45L	56.52 (< .001)	**1.45***	67.32 (< .001)	**1.57***	74.10 (< .001)	**1.60***
19	9R	107.30 (< .001)	**2.75***	114.77 (< .001)	**2.68***	105.24 (< .001)	**2.28***
20	45R	105.13 (< .001)	**2.69***	119.05 (< .001)	**2.78***	93.74 (< .001)	**2.03***
22	21L	-50.78 (< .001)	NA	-21.74 (< .001)	NA	-20.20 (< .001)	NA
24	21R	-50.05 (< .001)	NA	-26.31 (< .001)	NA	-21.85 (< .001)	NA
26	2L	29.95 (< .001)	NA	-17.95 (< .001)	NA	-10.21 (< .001)	NA
29	21L	3.23 (= .001)	NA	14.30 (< .001)	NA	17.08 (< .001)	NA
32	21R	-12.97 (< .001)	NA	-4.21 (< .001)	NA	-0.65 (= .518)	NA
35	17L	-37.62 (< .001)	NA	-45.14 (< .001)	NA	-47.35 (< .001)	NA
36	17R	-35.83 (< .001)	NA	-28.60 (< .001)	NA	-32.99 (< .001)	NA

Note. One-sample *t*-tests were used to detect the hemodynamic activations during the mindfulness meditation. * denotes that the channel was activated (i.e., *t*-value > 0, $p < .001$, *Cohen's d* > 0.5). NA indicates that the channel was not activated during meditation. L = left, R = right.

For Peer Review

Table S.6 Channel Activation Contrast between Two Meditations

Channel	Brodmann area	Session 1 (n = 1526) *t*-value (*p*)	Session 1 *Cohen's d*	Session 1 & 2 (n = 1832) *t*-value (*p*)	Session 1 & 2 *Cohen's d*	Session 1 & 2 & 3 (n = 2138) *t*-value (*p*)	Session 1 & 2 & 3 *Cohen's d*
1	11L	47.46 (< .001)	**1.27***	12.27 (< .001)	ND	11.88 (< .001)	ND
2	11R	48.44 (< .001)	**1.63***	16.52 (< .001)	**0.60***	14.84 (< .001)	ND
3	10L/R	48.87 (< .001)	**1.35***	17.92 (< .001)	**0.57***	17.22 (< .001)	ND
4	11R	58.70 (< .001)	**1.43***	33.65 (< .001)	**0.89***	32.92 (< .001)	**0.75***
5	46R	58.18 (< .001)	**1.57***	45.56 (< .001)	**1.21***	39.48 (< .001)	**1.02***
6	11L	74.08 (< .001)	**2.54***	34.75 (< .001)	**1.33***	32.70 (< .001)	**1.09***
7	46L	96.28 (< .001)	**2.58***	67.82 (< .001)	**1.76***	50.73 (< .001)	**1.38***
8	10R	44.45 (< .001)	**1.02***	15.30 (< .001)	ND	12.83 (< .001)	ND
9	10R	-4.81 (< .001)	ND	-16.27 (< .001)	ND	-18.69 (< .001)	ND
11	10L	67.65 (< .001)	**1.24***	23.17 (< .001)	**0.60***	23.92 (< .001)	**0.55***
12	10L	46.29 (< .001)	**0.76***	19.13 (< .001)	ND	18.22 (< .001)	ND
14	9L/R	5.76 (< .001)	ND	-8.08 (< .001)	ND	-9.96 (< .001)	ND
15	9R	27.13 (< .001)	**0.62***	12.92 (< .001)	ND	6.68 (< .001)	ND
16	9L	30.11 (< .001)	**0.67***	14.72 (< .001)	ND	4.55 (< .001)	ND
17	9L	98.99 (< .001)	**2.60***	39.54 (< .001)	**1.39***	40.00 (< .001)	**1.20***
18	45L	60.63 (< .001)	**1.61***	29.75 (< .001)	**0.90***	22.08 (< .001)	**0.62***
19	9R	-50.36 (< .001)	**-0.96***	-59.29 (< .001)	**-1.02***	-53.66 (< .001)	**-0.93***
20	45R	75.73 (< .001)	**2.21***	39.02 (< .001)	**1.16***	35.36 (< .001)	**0.89***
22	21L	32.99 (< .001)	**0.68***	24.99 (< .001)	NA	28.34 (< .001)	ND
24	21R	76.71 (< .001)	**1.58***	72.42 (< .001)	**1.17***	50.49 (< .001)	NA
26	2L	-36.43 (< .001)	**-0.94***	-25.20 (< .001)	NA	-30.22 (< .001)	ND
29	21L	23.86 (< .001)	**0.51***	21.85 (< .001)	ND	19.54 (< .001)	ND
32	21R	61.56 (< .001)	**1.36***	54.06 (< .001)	**1.13***	34.17 (< .001)	**0.81***
35	17L	4.96 (< .001)	NA	10.92 (< .001)	NA	5.93 (< .001)	NA
36	17R	23.49 (< .001)	NA	9.33 (< .001)	NA	5.99 (< .001)	NA

Note. Paired *t*-tests were conducted to compare the hemodynamic activations between intertemporal and mindfulness meditations. * indicates the activated channels with significant differences (i.e., $p < .001$, *Cohen's d* > 0.5), ND denotes non-significant results between meditation conditions. NA indicates that the channel was not activated during meditation. L = left, R = right.

For Peer Review

Table S.7 Channel Activation Contrast between Two Meditations in Experienced Participants

Channel	Brodmann area	Session 1 ($n = 1526$)		Session 1 & 2 ($n = 1832$)		Session 1 & 2 & 3 ($n = 2138$)	
		t-value (*p*)	*Cohen's d*	*t*-value (*p*)	*Cohen's d*	*t*-value (*p*)	*Cohen's d*
1	11L	162.76 (< .001)	**5.27***	80.35 (< .001)	**3.17***	63.74 (< .001)	**2.33***
2	11R	126.21 (< .001)	**4.86***	75.43 (< .001)	**2.92***	63.99 (< .001)	**2.22***
3	10L/R	129.03 (< .001)	**4.92***	70.93 (< .001)	**2.83***	59.30 (< .001)	**2.11***
4	11R	104.44 (< .001)	**4.71***	69.52 (< .001)	**2.97***	61.32 (< .001)	**2.34***
5	46R	123.05 (< .001)	**4.86***	76.24 (< .001)	**3.05***	66.06 (< .001)	**2.32***
6	11L	119.99 (< .001)	**4.94***	75.69 (< .001)	**3.06***	66.81 (< .001)	**2.38***
7	46L	127.80 (< .001)	**4.95***	79.54 (< .001)	**3.18***	64.43 (< .001)	**2.45***
8	10R	95.48 (< .001)	**3.24***	65.70 (< .001)	**2.03***	56.10 (< .001)	**1.47***
9	10R	89.31 (< .001)	**3.32***	60.86 (< .001)	**1.96***	46.16 (< .001)	**1.42***
11	10L	153.65 (< .001)	**3.62***	72.53 (< .001)	**2.44***	62.91 (< .001)	**1.97***
12	10L	100.46 (< .001)	**3.38***	67.68 (< .001)	**2.47***	52.21 (< .001)	**1.77***
14	9L/R	49.07 (< .001)	**1.57***	53.62 (< .001)	**1.63***	26.80 (< .001)	**0.86***
15	9R	-65.28 (< .001)	**-2.23***	-58.15 (< .001)	**-1.88***	-52.21 (< .001)	**-1.52***
16	9L	43.06 (< .001)	**1.70***	37.30 (< .001)	**1.18***	30.45 (< .001)	**0.93***
17	9L	66.64 (< .001)	**2.17***	42.56 (< .001)	**1.25***	32.77 (< .001)	**0.92***
18	45L	106.01 (< .001)	**3.96***	69.82 (< .001)	**2.74***	55.51 (< .001)	**1.95***
19	9R	74.13 (< .001)	**3.31***	55.12 (< .001)	**2.27***	42.37 (< .001)	**1.54***
20	45R	128.39 (< .001)	**5.55***	74.21 (< .001)	**2.99***	57.95 (< .001)	**1.96***
22	21L	113.82 (< .001)	**3.01***	56.77 (< .001)	**1.89***	60.65 (< .001)	**1.87***
24	21R	55.68 (< .001)	NA	43.56 (< .001)	NA	51.69 (< .001)	NA
26	2L	46.67 (< .001)	**1.80***	31.77 (< .001)	NA	27.49 (< .001)	NA
29	21L	32.48 (< .001)	NA	21.68 (< .001)	NA	17.74 (< .001)	NA
32	21R	206.77 (< .001)	**6.92***	92.49 (< .001)	**3.63***	75.02 (< .001)	**2.81***
35	17L	61.14 (< .001)	**2.33***	62.73 (< .001)	**1.82***	55.35 (< .001)	NA

Note. Paired *t*-tests were conducted to compare the hemodynamic activations between intertemporal and mindfulness meditations. * indicates the activated channels with significant differences (i.e., $p < .001$, *Cohen's d* > 0.5), ND denotes non-significant results between meditation conditions. NA indicates that the channel was not activated during meditation. L = left, R = right.

For Peer Review
